# Agricultural Trade Networks and Patterns of Economic Development

**DOI:** 10.1371/journal.pone.0039756

**Published:** 2012-07-02

**Authors:** Shade T. Shutters, Rachata Muneepeerakul

**Affiliations:** 1 School of Sustainability, Arizona State University, Tempe, Arizona, United States of America; 2 Center for Social Dynamics and Complexity, Arizona State University, Tempe, Arizona, United States of America; 3 Mathematical, Computational, and Modeling Sciences Center, Arizona State University, Tempe, Arizona, United States of America; Cinvestav-Merida, Mexico

## Abstract

International trade networks are manifestations of a complex combination of diverse underlying factors, both natural and social. Here we apply social network analytics to the international trade network of agricultural products to better understand the nature of this network and its relation to patterns of international development. Using a network tool known as triadic analysis we develop triad significance profiles for a series of agricultural commodities traded among countries. Results reveal a novel network “superfamily” combining properties of biological information processing networks and human social networks. To better understand this unique network signature, we examine in more detail the degree and triadic distributions within the trade network by country and commodity. Our results show that countries fall into two very distinct classes based on their triadic frequencies. Roughly 165 countries fall into one class while 18, all highly isolated with respect to international agricultural trade, fall into the other. Only Vietnam stands out as a unique case. Finally, we show that as a country becomes less isolated with respect to number of trading partners, the country's triadic signature follows a predictable trajectory that may correspond to a trajectory of development.

## Introduction

Network analysis has been increasingly used to disentangle and uncover patterns in a wide variety of complex systems, ranging from molecular (e.g., signal transduction pathways [1] to individual (e.g., social networks [2], [3]) to global (e.g., world city networks [4], [5]) scales. In this paper, we apply both contemporary and novel network analysis techniques to the global trade networks of agricultural products. While some analysis of trade networks has been conducted previously [6], [7], this study advances research on the topic through its foci on in-depth analysis of triads (three-node directed subgraphs) and on agricultural products.

We focus on agricultural products as they are necessary goods for all people, regardless of whether they reside in developing or developed countries. These goods are also responsible for substantial flows of virtual water between nations, significantly affecting a country's water footprint [8]. The trade networks of the agricultural products are subsets of the global trade web, which exhibits well-defined network characteristics [6], [9], [10]. Drivers of the topology of these trade networks include international politics, which affects the formation of trading partners, and heterogeneous environmental conditions, which constrain the ability of countries to produce certain agricultural products, thereby making these networks complex, important, and informative.

To better understand how trade networks vary across countries at different developmental stages and by traded agricultural products, we analyze the local structure of the trade networks, namely their triads. Although techniques of tabulating and analyzing triads in real networks has been used in social research for many years [11], a recent technique has been to compare the actual census of networks' triads to its expected frequencies. This technique has revealed that a small number of groups can describe a wide variety of real world networks, from gene transcription pathways to the world wide web [1], [12]. Here, we apply and extend this technique to the trade networks of agricultural products. This technique complements more traditional network analysis, which focuses on aggregate properties such as degree distribution, assortativeness/dissortativeness, or clustering patterns [13]. It focuses instead on local properties of the network topology and in particular we examine the ‘role’ that nodes play in their local triadic structures, not just whether they are a member of a triad or not. This analysis adds some ‘character’ to nodes, which has practical implications for understanding the status and development of different countries. To some extent this echoes a claim by Derudder and Witlox [4] that:

to understand the dynamics of ‘development’ in a given place, research should focus on how places are being transformed by their insertion in networks of commodities, knowledge, capital, labour, power, and how, at the same time, places and their institutional and social fabrics are transforming those networks as they locate in place-specific domains.

## Methods

We extracted national bilateral trade statistics for several agricultural commodities for the year 2000 from the online public databases of the Food and Agriculture Organization of the United Nations (faostat.fao.org, 2011). Data were modified as described in [10] (section 2.2). We used the existence of any level of trade to signify a directed trade link from the exporting country to the importing country; that is, our networks are not weighted. This is in contrast to [10], in which links were weighted by both the volume of trade and the water content of the commodity traded.

**Figure 1:**
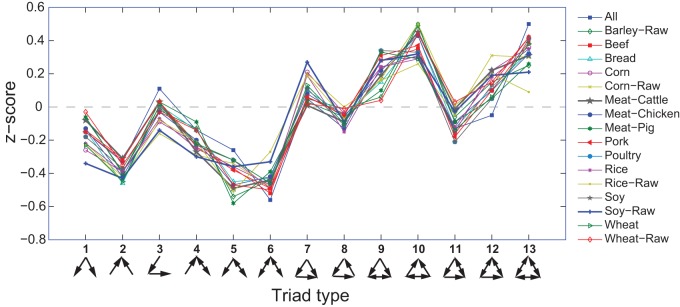
Triad significance profiles of agricultural trade networks: by product. These networks form a distinct superfamily not previously reported for other networks, including an international cargo shipping network.

Using this dataset, we first considered the overall topology of the full trade network by analyzing the degree distribution of both exports and imports and used Kolmogorov-Smirnov tests to determine whether those degree distributions statistically matched known distributions.

**Figure 2:**
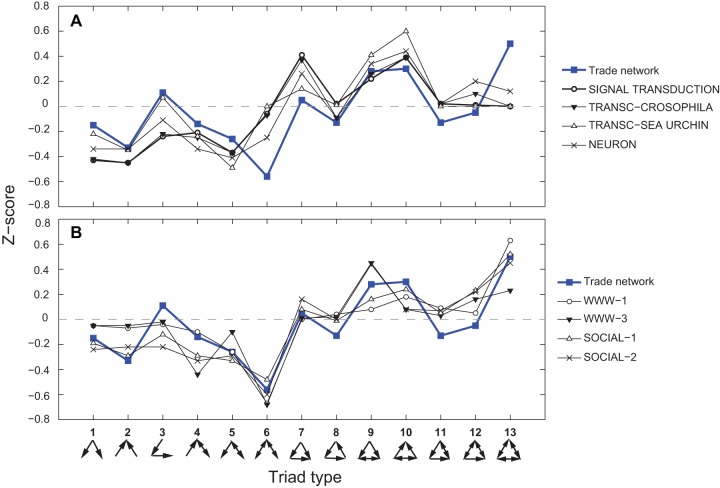
Comparison of the agricultural trade network TSP with known network superfamilies. The overall agricultural trade network compared to (A) biological regulatory networks and (B) human social networks. See Table 1 for correlation coefficients.

We then focus on the local structure of global agricultural trade networks by analyzing the frequency of different triads occurring in the networks. A triad is a connected subset, or subgraph, of three nodes within a network. Differences in the way that three nodes can be connected lead to 13 possible triad types in a directed network (bottom of Figure 1). Methods have long been used that involve tabulating the frequency of occurrence within a subject network of each of the 13 possible triad types and then making comparisons across networks [11]. A more recent methodological advance has been to compare the census of a network's triads to what would be expected from a randomly generated network [1], [12]. Frequencies of actual occurrence are compared to an expected frequency based on the how frequently they are observed in networks generated randomly while maintaining the same degree sequence. For each triad type this difference is then normalized and assigned a *z*-score:
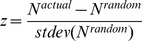



Graphing of *z*-scores versus triad type (e.g. Figure 1) gives the network's triad significance profile (TSP). Because *z*-scores are normalized and dimensionless, TSPs can be compared between networks governing vastly different systems regardless of differences in network size or density. Using this method Milo *et* *al.* 1] showed structural commonalities and differences across networks as varied as cellular signal transduction pathways, the world-wide web, personal acquaintance networks, and neural networks. Milo *et* *al.* grouped networks with highly similar TSPs, into what they call a network “superfamily.” We compared our overall trade network TSP with the two superfamilies that Milo *et* *al.* call biological regulatory networks and human social networks.

**Table 1:** Pearson pairwise correlations (R-values) comparing the triad significance profile (TSP) of the full agricultural trade network to TSPs of networks analyzed in [1] (Figure 1).

Biological information processing networks	
SIGNAL TRANS	0.5289
TRANSC-DROSOPHILA	0.5577
TRANSC-SEA URCHIN	0.6150
NEURONS	0.7301

Human social networks	
WWW-1	0.8676
WWW-3	0.7546
SOCIAL-1	0.8807
SOCIAL-2	0.8255

Note: See [1] for detail description of networks above.

Triads having a high positive *z*-score, meaning that the triad appears much more often than expected, are termed motifs of the network. For example, in the TSPs for agricultural trade networks presented in Figure 1, triads 7, 9, 10 and 13 would likely be considered motifs, depending on the product under consideration. Those triads having highly negative *z*-scores are termed anti-motifs.

**Figure 3:**
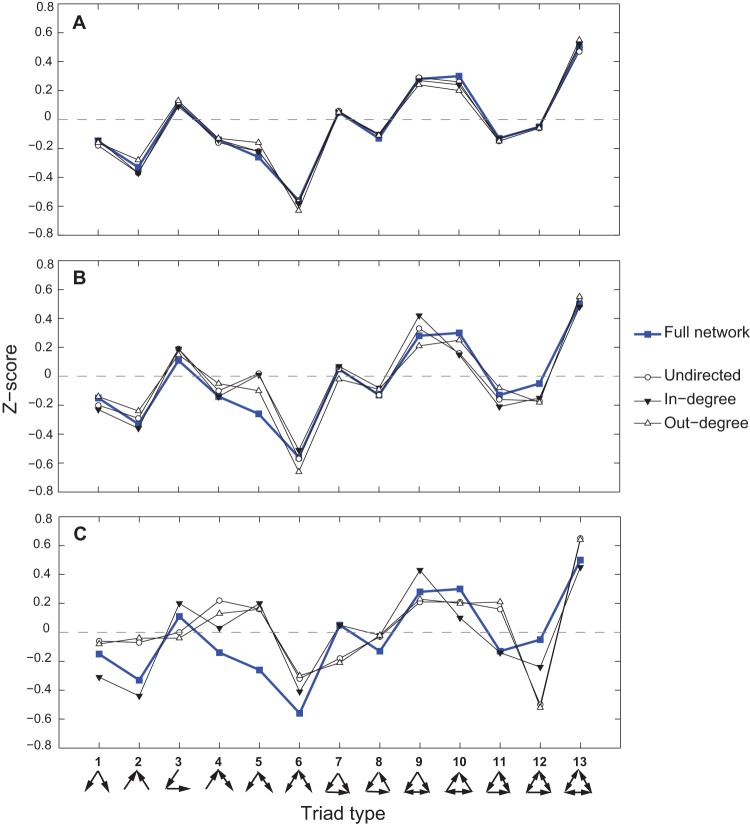
Robustness of the agricultural trade network to node deletion. Comparison of the TSP of the unaltered trade network to those of the same network when (A) 25%, (B) 50% and (C) 75% of the networks' more isolated nodes have been removed. Three methods of node removal included the nodes rank based on its in-degree, out-degree, and undirected degree. See Table 2 for correlation coefficients.

**Table 2:** Pearson pairwise correlations (R-values) comparing the triad significance profile (TSP) of the full unaltered trade network and TSPs of the network after node removal.

Basis of ranking nodes for removal			
Pct. of nodes removed	Undirected degree	In-degree	Out-degree
25%	0.9966	0.9950	0.9829
50%	0.9340	0.9238	0.9547
75%	0.6091	0.7929	0.5787

To assess the robustness of our TSP results to network perturbations, we removed large subsets of the full trade network and then reran the triadic analysis, comparing the resulting TSPs with the unaltered network's TSP. Because our trade links were not weighted we tested robustness by systematically removing sets of nodes before rerunning the triadic analysis. To determine which nodes to remove, we ranked nodes based on both in-degree and out-degree in the directed network, and by degree in the undirected version of the full trade network. We then removed 25%, 50%, and 75% of the least connected nodes from each of the three node lists before creating a new TSP. Differences between the TSPs of the original network and the nine versions of reduced networks were compared both graphically and numerically using Pearson correlations for each pairwise comparison.

**Figure 4:**
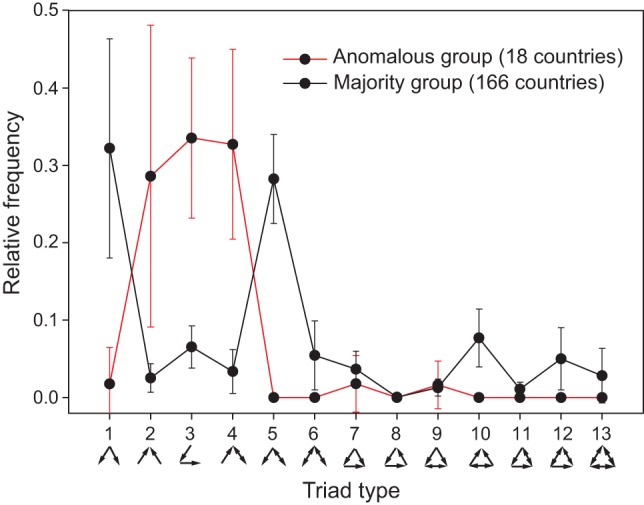
Triad distribution for two groups of countries. Most countries (166) share a similar triad distribution. However, 18 countries (listed in Table 3) differ distinctively from the majority group.

**Table 3:** Countries (18) whose trade network triad distributions deviate significantly from those of all other countries (166) and their global ranking in terms of isolation in the global agricultural trade network (i.e. Lesotho ranks as the least connected country).

Country	Trade isolation rank	Country	Trade isolation rank
Lesotho	1	Bhutan	10
Chad	2	Liberia	11
Guinea-Bissau	3	Mozambique	12
Tajikistan	4	Uzbekistan	13
Iraq	5	Afghanistan	14
Angola	6	Dominican Republic	17
Somalia	7	Laos	18
Haiti	8	Myanmar	21
Turkmenistan	9	Vietnam	138

Finally, we also examined in greater detail the roles that countries play in their local trade triads by analyzing their triad frequency distributions, as well as how these roles interplay with degree of connectedness.

## Results and Discussion

### Global network topology

We first consider the overall topology of the combined agricultural trade network by analyzing the degree distributions of both import and export links. We concur with the authors of [10] that the export degree distribution seemingly follows an exponential distribution, though we find that this is not statistically supported (Kolmogorov-Smirnov, h_0_: exponential distribution, d = 0.20018, p<0.01). One might also argue that the distribution qualitatively follows a power-law, lognormal, or other distribution with “fat tails”. Such distributions, or approximations of them, are typical of the scale-free architecture of regulatory networks. However, we find that the distribution of import degrees follows a normal distribution (Kolmogorov-Smirnov, h_0_: normal distribution, d = 0.05938, p  =  n.s.), which is more reminiscent of small-world networks typical in human societies [14].

**Figure 5:**
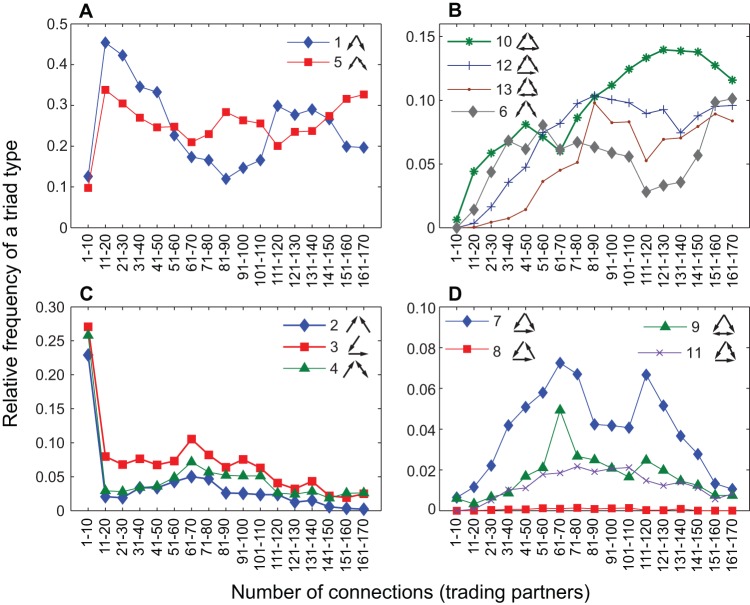
Relative frequency of each triad type as a function of degree of interconnectedness of the participant countries. Triad types are grouped into four figures based on their general trends and magnitudes. Note that type 8 (panel D) is consistently very infrequent, or almost non-existent: this is expected as type 8 represents three countries simply transporting food products in an endless cycle.

### Triad significance analysis and an agricultural trade superfamily

Construction of TSPs for the trade networks of several agricultural commodities as well as the full trade network, are presented in Figure 1. Our analysis reveals that TSPs for networks describing the global trade patterns for several agricultural products form a cohesive network superfamily distinct from those previously reported [1]. This is true even compared to the TSP exhibited by the international cargo shipping network [15], which adheres to the superfamily characterizing the world-wide web and human social networks.

**Figure 6:**
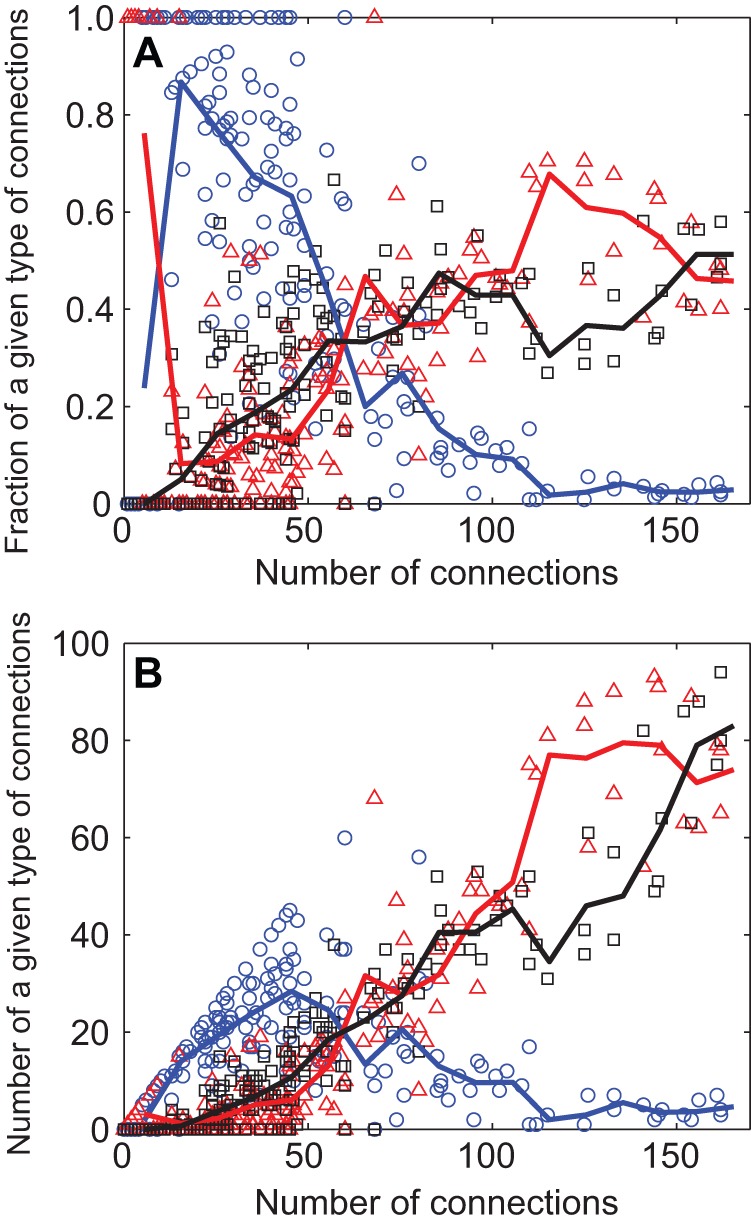
How countries allocate their different types of trade connections. The figure presents the number of trade links that are import only (blue), export only (red), and mutual (black) vs. total links, i.e., the number of trade partners or degree of interconnectedness.

When compared to networks analyzed in [1], agricultural trade networks appear to share similarities with both the superfamilies of biological information processing networks (motifs 9 and 10) and human social networks (motif 13 and anti-motif 6). Motif 13, which is the hallmark of human social networks [1], is especially prominent in the TSPs of all agricultural commodities. Figure 2 presents a visual comparison of the overall agricultural trade network with those analyzed in [1]. Pearson correlations between the agricultural trade network TSP and those analyzed in [1] (Table 1) are stronger for human social networks (mean R = 0.83) than for biological networks (mean R = 0.61). Given the finding above that the aggregate network topology exhibits an export degree distribution typical of regulatory networks and an import distribution typical of small-world networks, it is to some extent consistent that the overall TSP displays a combination of both biological regulatory networks and human social networks.

We speculate that the existence of this unique superfamily is partly the result of the complex interplay between geography, climate, and politics. Superfamilies thus far uncovered are based on networks that are either a function of geographical proximity only or have no relation to geography at all. However, proximity alone does not capture the heterogeneity of climate, politics, and culture that are exhibited by countries comprising the global trade networks for agricultural products and this mixture of biophysical and social factors may be responsible for the emergence of the distinct superfamily.

In addition, certain products, such as wheat and barley, exhibit some features different from other food commodities, namely triad 10 being more pronounced and triad 9 being significantly suppressed. It should also be noted that the aggregate network (in which a connection between two countries is considered established if at least one type of agricultural product is traded between the two) has the highest *z*-score for triad 13. This is not surprising. Given that this profile is derived from the aggregate network of several products, one would expect it to be the most densely connected.

In addition, in the aggregate TSP for all agricultural products, triad 6 had the lowest *z*-score, meaning that it occurred rarely compared to the frequency expected in the randomized networks. Interestingly, this particular triad is one that Facchettie *et* *al*. [16] refer to as unbalanced (sometimes called the ‘frustration’ configuration) because the middle node has positive relationships with two partners who are not connected and may have a negative disposition towards one another. The low *z*-score for triad 6 supports a long held view of social networks known as the structural balance theory [17], which asserts that unbalanced triads like triad 6 will tend to be underrepresented in human social networks as a way of avoiding potential conflict.

### Robustness of the triadic analysis

When removing large subsets of more isolated nodes from the full trade network as described above, our results remain quite robust (Figure 3). Pearson pairwise correlations are presented in Table 2 comparing the unaltered network before and after various treatments of node deletion. Removing the 25% of least connected nodes resulted in almost no difference in TSP (Figure 3A). When half the network's nodes were removed, resulting TSPs (Figure 3B) remained largely unchanged (Pearson correlations, R>0.92 in all cases). With 75% of nodes removed, TSPs begin to look qualitatively different (Figure 3C), though correlations remain relatively high (Pearsons correlation, 0.58>R>0.79).

This result indicates that the TSP for the global agricultural trade network is relatively stable and is likely robust to large and sudden changes in global trade patterns. It should be noted that this conclusion is in regard to the local-scale *topological* structure of the trade network only and one should not conclude that individual countries or the global economy would be unaffected by such disruptions in trading patterns.

### Trade isolation vs. simple triad distribution

The TSPs presented above offer a helpful tool for classifying and comparing networks across a broad range of complex systems. Once candidate networks of a superfamily are identified, insights from one member network may be transferred to others. However, such analysis alone does not offer much in the way of explaining *why* such superfamilies have evolved or how superfamily members might be related (but see [18] for a promising method of explaining evolutionary origins of some network superfamilies). One reason is that by comparing actual triad frequencies with those in a randomized network with the same degree sequence and the same numbers of directed and mutual edges, as in [1], the analysis does not consider why the network has its particular degree sequence and edges in the first place. Especially in this case, where trade networks emerge as a distinct superfamily, we must examine their network structures at a more fundamental level to obtain a better understanding.

To this end, we first consider each country's simple triad frequency distribution. That is, for each country, all triads to which it belongs are classified into one of the 13 triad types, and the relative frequency of each triad type calculated. This is in contrast to the normalized frequency relative to that of a randomized network (sensu [1]) that generates the TSPs above. We then group countries by their degrees, or numbers of trade partners, including all three types: import, export, and mutual. Of the 184 countries included in the aggregate network, 166 display a remarkable similarity in their triad distributions with triads 1 and 5 generally being most frequent (Figure 4). However, 18 countries (Table 3) exhibit a distinctly different distribution with triads 1 and 5 being highly infrequent or non-existent, and triads 2, 3, and 4 being very frequent (Figure 4).

Our initial hypothesis regarding countries deviating from the dominant triad distribution was that these deviating countries lagged other countries in some metric of development (i.e. industrialization, trade integration, market infrastructure) or environmental impact (carbon footprint, ecological footprint). However, a review of the deviating countries found no correlation between these countries and the United Nations Human Development Index, membership in the World Trade Organization, nominal classification as a 3^rd^ world country, or trade-based carbon footprint. Although most members of the deviating group do share a degree of underdevelopment, many other countries that would also be considered developmentally similar fit neatly into the dominant profile (e.g. Malawi, Cambodia, and Gambia, among others).

Our analysis does show that these deviating countries share substantially one attribute – low connectedness to the global agricultural trade network. Table 3 reveals that of the 18 deviating countries, 17 rank among 21 least connected, or most isolated, countries with respect to agricultural trade links. Other countries that rank high in terms of isolation, but still conform to the dominant profile, are predominantly small island countries (Cook Islands 15^th^, Kiribati 16^th^, Tonga 19^th^, Solomon Islands 20^th^, Montserrat 23^rd^, Faroe Islands 24^th^).

Figure 5 exhibits the relative frequency of each triad type as a function of country degree or connectedness. The results show that as countries leave the most isolated group and enter the less isolated ones, the changes in their triad profiles are marked primarily by sharp rises of triad types 1 and 5 and sharp drops in triad types 2, 3, and 4.

### Subtriadic analysis: exporter, importer or trade facilitator?

To understand these patterns, one needs to look not only at the triad types in which a country participates but also what role it plays within that triad. Accordingly, Figure 6 shows how the three types of edges, or trade connections – import, export, and mutual – are allocated as countries become more and more connected.

The general trend is as follows. Countries seek a trade surplus through increased exports but must follow a trajectory through different phases of economic interconnectedness. As a country increasingly engages in global trade, it develops a demand for goods that must be imported from elsewhere. This continues until a country establishes approximately 50 trade partners. At that point the country has sufficiently developed to produce goods and export them to other countries, both new countries and those who are already their trade partners. This increases the country's export edges and converts import edges into mutual links (Figure 6b). This increasing trend of export continues as countries become more integrated into the global trade network.

**Figure 7:**
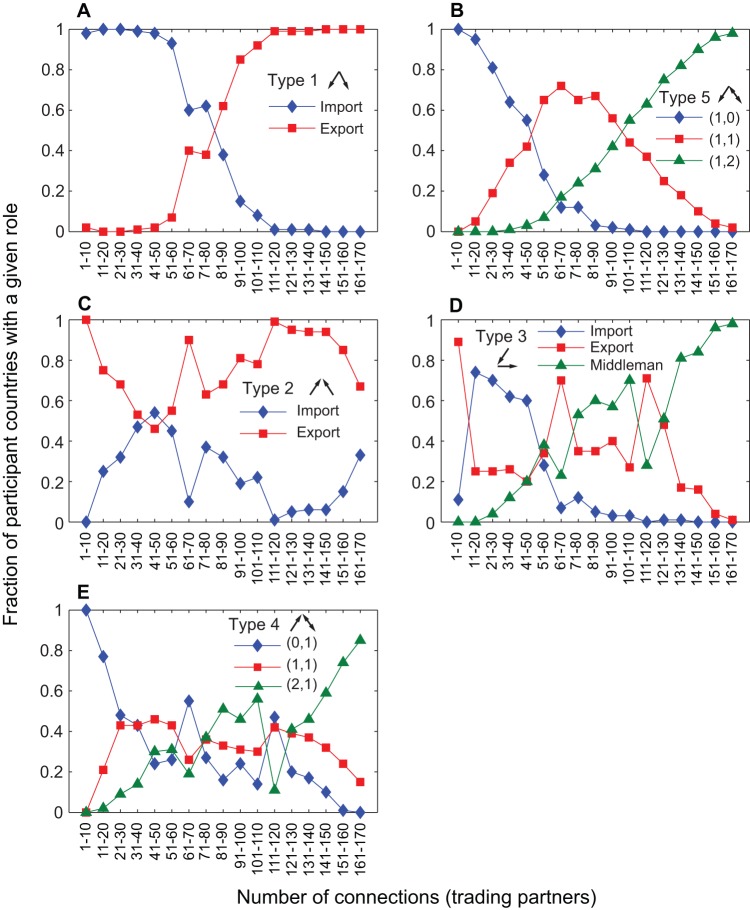
Fraction of participant countries with different roles in a given triad type as a function of their interconnectedness. In cases where the roles cannot be clearly named, we simply report the in and out degrees of the node in the format (in, out). The y-axis in this figure can be interpreted as a conditional probability, namely, the probability that a country plays a certain role *given* the triad type and the country's connectedness.

This is consistent with patterns shown in Figure 7, which considers the frequency of distinct roles played by countries in a given triad type. For a given triad 1, agricultural products tend to flow from more connected (more developed?) countries to those that are more isolated. For a given triad 5, the most connected country (with (in, out)  =  (1,2)) tends to link up its two other less connected partners, exporting to the more isolated one and exchanging products with the more connected. In contrast, the only somewhat consistent trend for triads 2, 3, and 4 is that more connected countries tend to play the role of a “connector” linking two more isolated partners, a pattern similar to triad 5. The lack of clear trends for import and export links for these types suggests the possible signature of a transition period during which countries experiment with different trading partners. At the same time, large fractions of triads 1, 5, 6, 10, 12, and 13 seem to represent more mature economies (Figure 5).

### Bilateral trade of a single commodity

An interesting aspect of the individual commodity trade networks is the existence of bilateral trade of a single good, meaning that country A exports a commodity to country B while country B exports the same commodity to country A. While two nations exporting to each other in the overall trade network is expected, it is not clear why two countries would export the same commodity to each other. Yet such bilateral trade relationships are an integral part of many of the over-represented triads, particularly triads 9, 10, 12, and 13 (Figure 1). In fact, 18.4% of all pairwise relationships in the various individual commodity networks are bilateral.

Our data as supplied by the FAO have no further detail on what constitutes the goods within each FAO category. However, we offer three plausible explanations for the relatively high incidence of bilateral trade links for a given commodity:

1. Different breeds or varieties of goods are consolidated in the FAO groupings. For instance, country A may export Indica rice to country B, while country B exports aromatic rice to country A. Because the FAO classification scheme does not distinguish among varieties, this would appear as bilateral trade of rice.
2. Seasonal differences. Countries A and B may have different growing seasons for the exact same agricultural commodity so that trade flows from A to B part of the year, and from B to A in other parts of the year.
3. Geographical proximity to foreign markets. In a hypothetical example, assume the only two suppliers in North America for a certain commodity are in Vancouver, Canada, and Boston, USA. Because of proximity and transportation costs, the supplier in Vancouver may supply most of the Western USA while the supplier in Boston supplies most of Eastern Canada. Again this would appear as bilateral trade of the hypothetical commodity.

### The curious case of Viet Nam

Analysis by country of local structure in the world agricultural trade network revealed that Viet Nam alone did not fit neatly into the two groupings described above. Though its triad distribution essentially matches that of the isolated group, Viet Nam is nevertheless quite well connected with respect to agricultural trade. It ranks near the top quartile of countries *most* connected to the world trade network for agricultural products. One possible explanation is that, despite its status as a rapidly developing market and its large number of trading partners, its trade policies have affected its transition to the majority distribution group. This speculation is somewhat supported by Athukorala's 19] claim that Viet Nam's protectionist trade policies make the country out of step with other major trading nations. If this holds true, the triadic analysis methods used in this study may be a useful tool for policy makers by revealing whether their policies are facilitating or hindering integration into the world trade network.

### Final remarks

In summary we have applied and extended triadic network analysis to the global trade networks of agricultural products. Results show that such networks exhibit a distinct triad significance profile (TSP), or “superfamily,” distinguishable from other networks reported thus far by its combination of elements from biological regulatory networks and human social networks. A more fundamental analysis of triad distribution indicates that relatively isolated and connected countries engage in very different configurations of triads. Furthermore, the roles played by the countries (e.g., importer, exporter, or trade facilitator) in a given triad type change with their interconnectedness, which could potentially be indicative of their economic developmental stages.

We anticipate that both findings and methods reported herein should contribute to understanding this and other types of global networks. One promising potential contribution is the application of this method to trade networks with cities as nodes. The differences and similarities across spatial scales will potentially lead to the transferability of knowledge and understanding from one scale to another. In addition, analyzing the same network at different times could further contribute to supporting or refuting some of the conjectures presented in this paper.
